# PathRings: a web-based tool for exploration of ortholog and expression data in biological pathways

**DOI:** 10.1186/s12859-015-0585-1

**Published:** 2015-05-19

**Authors:** Yongnan Zhu, Liang Sun, Alexander Garbarino, Carl Schmidt, Jinglong Fang, Jian Chen

**Affiliations:** Department of Computer Science and Electrical Engineering, University of Maryland, Baltimore County, 1000 Hilltop Circle, Baltimore, MD USA; Department of Computer Science, Hangzhou Dianzi University, Hangzhou, Zhejiang Province P.R. China; Department of Animal & Food Sciences, University of Delaware, Newark, DE USA

## Abstract

**Background:**

High-throughput methods are generating biological data on a vast scale. In many instances, genomic, transcriptomic, and proteomic data must be interpreted in the context of signaling and metabolic pathways to yield testable hypotheses. Since humans can interpret visual information rapidly, a means for interactive visual exploration that lets biologists interpret such data in a comprehensive and exploratory manner would be invaluable. However, humans have limited memory capacity. Current visualization tools have limited viewing and manipulation capabilities to address complex data analysis problems, and visual exploratory tools are needed to reduce the high mental workload imposed on biologists.

**Results:**

We present PathRings, a new interactive web-based, scalable biological pathway visualization tool for biologists to explore and interpret biological pathways. PathRings integrates metabolic and signaling pathways from Reactome in a single compound graph visualization, and uses color to highlight genes and pathways affected by input data. Pathways are available for multiple species and analysis of user-defined species or input is also possible. PathRings permits an overview of the impact of gene expression data on all pathways to facilitate visual pattern finding. Detailed pathways information can be opened in new visualizations while maintaining the overview, that form a visual exploration provenance. A dynamic multi-view bubbles interface is designed to support biologists’ analytical tasks by letting users construct incremental views that further reflect biologists’ analytical process. This approach decomposes complex tasks into simpler ones and automates multi-view management.

**Conclusions:**

PathRings has been designed to accommodate interactive visual analysis of experimental data in the context of pathways defined by Reactome. Our new approach to interface design can effectively support comparative tasks over substantially larger collection than existing tools. The dynamic interaction among multi-view dataset visualization improves the data exploration. PathRings is available free at http://raven.anr.udel.edu/~sunliang/PathRings and the source code is hosted on Github: https://github.com/ivcl/PathRings.

**Electronic supplementary material:**

The online version of this article (doi:10.1186/s12859-015-0585-1) contains supplementary material, which is available to authorized users.

## Background

Biology has entered an era when our ability to collect data has outstripped our ability to turn that data into knowledge. In particular, high-throughput sequencing is providing enormous amounts of information about gene-expression patterns in a large number of species. In many cases, the experimental objective is to compare two or more distinct biological states, such as disease and control, in order to understand the ramifications of changes in gene expression. Typically these high-throughput data are interpreted in the context of signaling and metabolic pathways, and several resources are available that warehouse and provide visualization tools for pathway analysis (e.g., [1-3]). Visualization is a valuable way to assist in pathway exploration by rendering large amounts of data, thereby aiding investigators’ ability to generate testable hypotheses and knowledge.

Some existing tools either list overlaps between datasets, such as Lists2Networks [4] and Kerfuffle [5], or overlay information onto the individual pathway graph network, such as BioVenn [6], VisANT [7], CHIBE2 [8], and MIMO [9]. These tools are useful in identifying the interconnections between multiple datasets, but they lack a general overview of all pathways. Such an overview would be valuable in rapidly interpreting the global implications of changes in gene expression patterns and to developing a general view of differences among multiple biological states.

Other tools provide an overview for pathway relationships by overlaying information onto pathways, e.g., iPath gives an overview of regulatory pathways [10]. However, manual intervention is still required to identify interesting pathways. Cellular overview provides organism-specific metabolic map diagrams, but additional information such as expression data is still displayed in a single pathway diagram [11]. Many pathway databases such as Reactome use a tree view to list the hierarchical pathways, but no high-level overview is available to place experimental results within the context of a large biological network.

Most existing tools are restricted to presenting visualization results in a single view at a time, limiting data comparison [12] and forcing humans to store information in their working memory during analysis process. Additionally, displaying data is limited by the physical size of the screen [13]. Some tools, such as Reactome [1] and Cell Overview [11], use a zoomable user interface that lets users navigate in a fixed view; however, this approach does not support multiple and complex dataset comparison.

**Figure 1:**
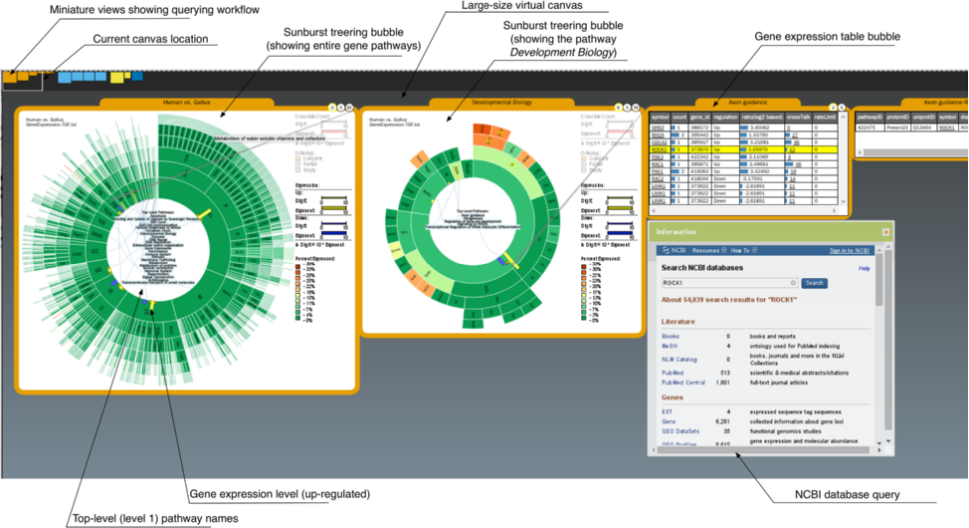
PathRings interface.

Here we present PathRings, a web-based framework for interactive exploration of biological pathway networks (Figure 1). Its features include: (1) **Bubbles views**: applying the ideas of VisBubbles [13], PathRings extends traditional static multiple views to a bubble-based interface. Bubbles do not overlap, but can be grouped and ungrouped, which helps users perform dynamic analysis and switch views for comparative studies. (2) **Virtual working space**: PathRings applies the idea of “virtual screen space extension” [13] by providing a panning navigation bar at the top of the window and extending the current view space to a much larger continuous working space. (3) **Sunburst compound graph visualization**: PathRings displays a hierarchical view of all human pathways from Reactome [1] in a single sunburst visualization, and each arc of a sunburst view is a unit of query and can be dragged out to form a new sunburst compound graph in a new view for sub-pathway analysis. Gene expression, crosstalk and ortholog data can be overlaid. Our exploration workflow supports overview to detail exploration: while sunburst visualization can give an overview of all the pathways, it also displays detailed sub-pathway information in a greater scale. The extendable interface can create new detailed views and support a comparison of multiple experimental data.

PathRings attempts to solve several challenges in pathway visualization that vary depending upon users’ exploration goals. At one level, comparing pathways between species allows insight into evolution. Such comparison requires identification of orthologous sequences between a reference and a target genome. PathRings uses Human Reactome as the reference to predict pathways in other species. Interspecies comparisons are then displayed that depict pathway differences between the two species. A second usage scenario is to evaluate gene or protein expression data from a single species in the context pathways. One complexity that current visualizations rarely handle is that individual gene products can affect multiple pathways. Changes in the expression of such cross-talking gene products may affect multiple pathways and have significantly more impact on biology than gene products that function in a single pathway. In addition, few current visualization schemes indicate rate-limiting gene products in the context of pathways. PathRings addresses these issues by identifying both cross-talking and rate-limiting gene products in the context of pathways.

## Implementation

PathRings is written in JavaScript Language and HTML5. The main bubble-based interface framework was developed in HTML5 two-dimensional (2D) Canvas API, while the Sunburst visualization is implemented in D3 [14]. It uses Ajax to get data from the server along with MySQL databases to manage the biological pathway data. PHP is used for extracting data from the server and MySQL databases. PathRings is designed to be easily extensible and allows developers to create new features for interactive data analysis.

## Results

### Pathway generation

We have extended the Reactome human pathways [1] to support cross-species analysis. PathRings supports the analysis of human, mouse, chicken, alligator, and turtle gene expression data. Here the mouse, chicken, alligator, and turtle pathways are predicted based on orthologous relationships between the human and target genomes, thus our pathways are more complete than Reactome. Investigators can use PathRings for other species when an orthology mapping is available between the targeted species and those of human gene products. For chicken Reactome, pathways have been augmented by including orthologous genes identified by RNAseq analysis (Schmidt, unpublished). Hence, we refer to the chicken Reactome as Gallus Reactome Plus.

### PathRings: an overview

PathRings’ sunburst visualization can depict the impact of the expression data on all Reactome pathways. The user can select interesting pathways for further analysis by clicking a certain arc of the sunburst to create a new sunburst bubble visualization to examine sub-pathways or to create a gene table bubble visualization listing all the affected genes (Figure 1). The user can select a symbol name in the table to obtain gene information from NCBI [15].

Comparison of multiple pathways can be made by grouping concurrent views and editing one view will affect the other view. No analysis will need to be deleted and moving the current canvas location on the panning bar to another view (or analytical process), while keeping the previous exploratory analysis in context (Figure 1).

PathRings supports pathway exploration for the four types of relationships between pathway members: hierarchical relationships, cross-talking relationships, orthologous relationships, and gene expression relationships.

### Hierarchical relationships between pathways

**Figure 2:**
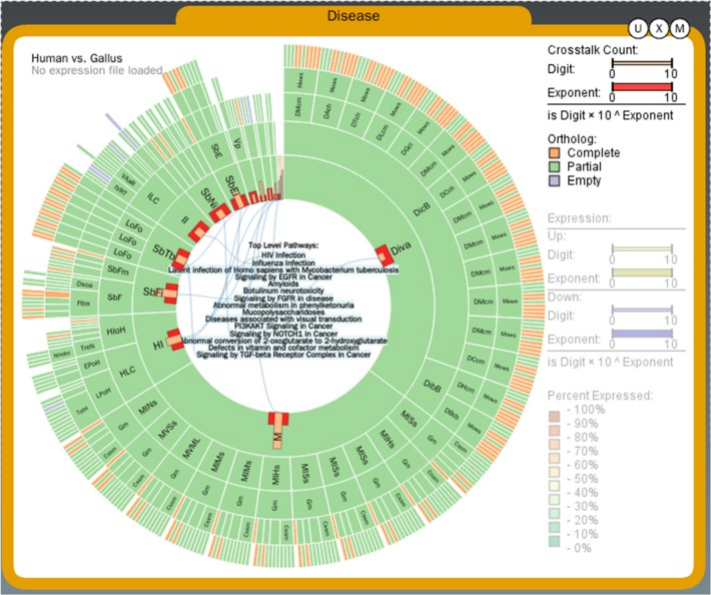
Pathway hierarchical relationships.

Reactome provides a hierarchical depiction of pathways by dividing large pathways into multiple sub-pathways in a tree structure, depicted in a Sunburst view (Figure 2). Sunburst represents the hierarchical structure in a radial layout to confine information in a space, useful for visualizing mid-sized trees [16] while keeping an intuitive hierarchical structure [17]. In PathRings, every arc of the sunburst shows a parent-children pathway relationship, and overlaying experimental data on the pathways facilitates concurrent exploration.

Here the visualized gene expression level is represented using the order of magnitude markers approach (OOMM) to fit the large-dynamic range expression data [18]. An expression level is represented using the scientific notation of A × 10^B^, where A is the digit (a real) and B is the exponent (an integer). The integer exponent B uses a wider bar and the real digit A is shown using a narrow bar. Both A and B are on a linear scale from 0 to 10. For example, an expression level of 99 will be re-written as 9.9 × 10^1^, and the wider bar showing 1 whilst the narrow bar showing 9.9. In this way, both large and small expression values can be precisely perceived.

### Cross-talking relationships between pathways

**Figure 3:**
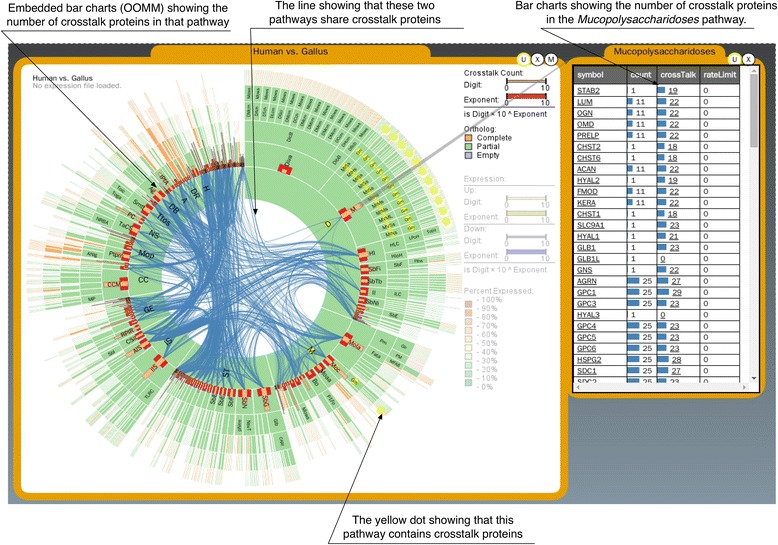
Cross-talking relationships between pathways. This example shows cross-talking at level two in the pathway hierarchy. The OOMMs represent the number of shared proteins of two species (here human and gallus).

In biological pathways, biological crosstalk refers to instances in which one or more components of one pathway affects another; and such crosstalk is often described using cross-talking gene products, as an interconnection relationship between two or more pathways [19]. PathRings shows a cross-talking relationship between pathways by edge links (Figure 3). Details about cross-talking gene products are listed in a table when needed showing the experimental data analysis process. Users can still click the cross-talking gene from a crosstalk table to highlight the cross-talking pathways marked in yellow dots. The number of crosstalk genes is also represented using the OOMM approach.

### Orthologous relationship between Species

**Figure 4:**
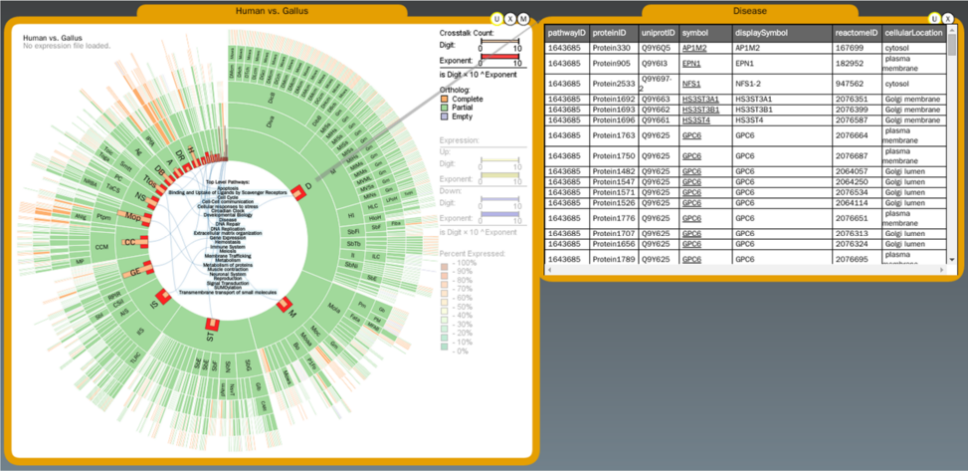
Orthologous relationships between species. Ortholog is complete or partial or empty. Users can query the information of the ortholog genes by clicking the OOMM bars and a table bubble will display the ortholog table (here human and gallus).

Pathway prediction based on orthology between a reference genome (human for Reactome) and a target (such as chicken) will yield three types of orthologous pathway relationships between the reference and target species: complete, partial, and empty defined as the following (Figure 4).

- Complete: All genes in a pathway can be identified in a target species based on orthology to the reference.
- Partial: Not all genes in a pathway are present in the target species.
- Empty: No orthology is present in the target species.

In PathRings, all three relationships of a pathway are encoded by color overlaying the arc of the sunburst (green, yellow, and purple for complete, partial, and empty accordingly). The default view of the sunburst shows the orthologous relationships between human and chicken. Other species of mouse, alligator, and turtle can be loaded for further analysis.

We provide an overview of orthologous genes (shared proteins) of a pathway between two species by embedding bar charts on each arc of sunburst. The height of the bar corresponds to the number of orthologous gene, so that the user can easily find the interesting pathways. Orthologous genes can be queried and listed in a table that includes gene symbol, the number of gene products in the pathway, the number of cross-talking pathways influenced by this gene and the gene product’s rate-limiting status all shown in bar charts in different columns (Figure 4). The user can reorder the table by clicking the header of each column to search for data of interests, get detailed gene product information, and see the cross-talking relationship. Views are linked such that clicking on the cross-talking gene (highlighted in yellow in the table) will highlight the cross-talking pathways that contains these genes in the sunburst view in large yellow dots. Finally, the user can also open two or more sunburst bubbles and load their ortholog data for comparative analysis.

### Visualization of gene expression information

**Figure 5:**
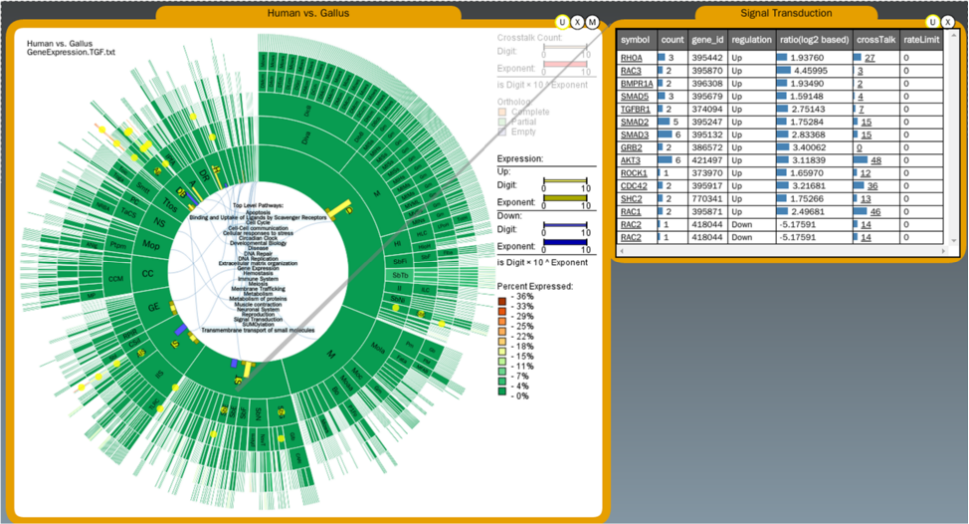
Gene expression data. Differentially expressed gene sets are color-coded. OOMMs show the number of differentially expressed genes and yellow for up-expressed genes and blue for down-expressed genes. User can query the gene products in a specific pathway by clicking the OOMM bars and a table bubble will display the gene product table.

PathRings supports the display of gene expression data from experiments or in controlled conditions (Figure 5). Gene expression levels, calculated from existing orthology mappings [20] color pathways in the sunburst. Users can analyze their gene expression data from different biological states according to the Entrez gene identification number, the gene symbol, and the ratio of the expression level. The user may input a cutoff threshold value for analysis or just use the default value. Color is used to encode the ratio between differentially expressed gene sets (sum of up-expressed genes and down-expressed genes) and the total number of orthologous proteins of each pathway, providing an overview of the gene expression relationships in all pathways. OOMMs are embedded on the sunburst to show the number of deferentially expressed gene sets. To identify the genes affecting a pathway, the user can click the pathway to retrieve a table that includes the Entrez gene identification number, gene symbol, number of gene products in the pathway, log-2-based ratio of each symbol, regulation status (up and down), number of cross-talking pathways influenced by this gene, and the gene product’s rate-limiting status.

## Discussion

### Species consideration

Our current implementation only supports a few species. Our long-term vision is to integrate this exploratory analysis to iPlant infrastructure [21] thereby making several thousand species accessible to the community.

### Exploration vs. automatic statistical approach

Automated methods play a central role in biological pathway analysis. For example, we could potentially use Gene Set Enrichment Analysis [22] for interpreting gene expression data by assigning a score to a pathway through a statistical analysis to measure similarities in pathways. However, many other features might be difficult to identify through automated methods alone. Visualization can help exploration especially when performing unstructured browsing and locating tasks or pre-filtering a set of entities. Broadly speaking, no visualization tools exist for comparing large sampling data that scale beyond small stretches of several pathways. Even current tools, such as Reactome could be scaled to several pathways, we find that the design often did not allow for large scale comparisons and focused explorations thus is lack of visual scalability.

## Conclusions

PathRings lets biologists explore pathway datasets in a dynamic fashion that pathway hierarchies, ortholog, crosstalk, and NCBI are integrated to answer biological questions. It provides an overview of all the pathways for analyzing experimental data and supports uploading experimental data. Novel visual interface design supports rapid visual retrieval and comparative exploration for efficient data inspection.

### Availability and requirements

- **Project Name:** ABI Development: PathBubbles for Dynamic Visualization and Integration of Biological Information
- **Project home page:**https://sites.google.com/a/umbc.edu/pathbubbles/
- **Operating system(s):** Platform independent
- **Programming language:** HTML, Javascript, PHP
- **Other requirements:** Web browser
- **License:** BSD license
- **Any restrictions to use by non-academics:** no restriction
